# Semaglutide in peripheral artery disease and diabetes by baseline disease severity and age: the STRIDE trial

**DOI:** 10.1093/eurheartj/ehag129

**Published:** 2026-03-05

**Authors:** Joakim Nordanstig, Ecenur Guder Arslan, Andrei-Mircea Catarig, Kim Houlind, Bernhard Ludvik, Neda Rasouli, Harald Sourij, Sebastian Thomas, Subodh Verma, Marc P Bonaca

**Affiliations:** Institute of Medicine, Department of Molecular and Clinical Medicine, University of Gothenburg, 41345 Gothenburg, Sweden; Novo Nordisk A/S, Søborg, Denmark; Novo Nordisk A/S, Søborg, Denmark; Horsens Regional Hospital, Denmark; Department of Regional Health Research, University of Southern Denmark, Odense, Denmark; 1st Medical Department and Karl Landsteiner Institute of Obesity and Metabolic Disorders, Landstrasse Clinic, Vienna, Austria; Division of Endocrinology, Metabolism and Diabetes, University of Colorado School of Medicine, Aurora, CO, USA; Cardiometabolic Trials Unit, Division of Endocrinology and Diabetology, Medical University of Graz, Graz, Austria; Novo Nordisk GBS India, Bangalore, India; Division of Cardiovascular Surgery, St. Michael’s Hospital and University of Toronto, Toronto, ON, Canada; CPC Clinical Research and Division of Cardiology, University of Colorado School of Medicine, Aurora, CO 80045-7120, USA

## Introduction

Walking limitation and claudication are defining features of peripheral artery disease (PAD), and common in persons with type 2 diabetes (T2D). Despite this burden, effective therapies for PAD remain scarce. In T2D, assessment of PAD severity is challenging as the multifactorial nature of limb symptoms and the frequent presence of atypical presentations obscure associations with functional outcomes.^1^

The STRIDE trial demonstrated efficacy of semaglutide for improving functional capacity in people with Fontaine IIa PAD and T2D.^2^ Pharmaceutical therapies for claudication may however vary in efficacy across clinical markers of PAD severity.^3^ Clinical history of peripheral revascularization procedures (that structurally change vessels) or presence of polyvascular disease both indicate a more progressed PAD given their association with subsequent limb and vascular events.^4,5^ Impaired renal function may also act as a marker of PAD severity, reflecting well-established pathological effects on vascular biology associated with hypoalbuminaemia, chronic inflammation, and the pro-calcific state of chronic kidney disease,^6^ with trial data suggesting semaglutide efficacy may interact with baseline estimated glomerular filtration rates (eGFR).^7^ Finally, polypharmacy and pharmacokinetic changes in advanced age may modify medication efficacy, but these patient populations continue to be underrepresented in clinical trials.^8^

In this *post hoc* analysis from STRIDE, we investigated the consistency of semaglutide’s efficacy across these markers of PAD severity and age.

## Methods

The STRIDE trial was a randomized, double-blind, placebo-controlled trial (NCT04560998), comparing once-weekly sub-cutaneous semaglutide 1.0 mg to a matched placebo in adults with PAD and T2D with intermittent claudication.^2^ Primary and confirmatory secondary endpoints were ratio-to-baseline in maximum walking distance (MWD) and pain-free walking distance (PFWD), respectively, on a constant load treadmill (3.2 km/h, 12% inclination) at week 52.

This *post hoc* analysis assessed MWD and PFWD across subgroups associated with PAD severity [eGFR (</≥60 mL/min/1.73 m^2^)], peripheral revascularization history, polyvascular disease, and age (</≥75 years). Polyvascular disease was defined as having either coronary heart disease (prior myocardial infarction, coronary revascularization, and/or ≥50% stenosis in coronary artery) and/or cerebrovascular disease (prior stroke, carotid artery revascularization, and/or ≥50% stenosis in carotid artery).

The analysis employed the trial product estimand using the on-treatment without rescue treatment observation period, evaluating treatment effect in all randomized participants under the assumption that they remained on the study product and did not initiate rescue treatment. Post-rescue data were excluded from analysis. Post-baseline log-transformed data at week 52 were analysed using a mixed model for repeated measurements with an unstructured covariance matrix employed for within-participant measurements. The model included treatment, region and subgroup as fixed factors, a treatment-by-subgroup interaction term, and baseline value of the endpoint as a covariate, all nested within visit. Treatment effects were described by estimated treatment ratios (ETRs), defined as the ratio of the estimated geometric mean ratio-to-baseline of the endpoint for semaglutide vs placebo. Interactions were tested at a 5% significance level, not adjusted for multiplicity.

## Results

**Figure 1:**
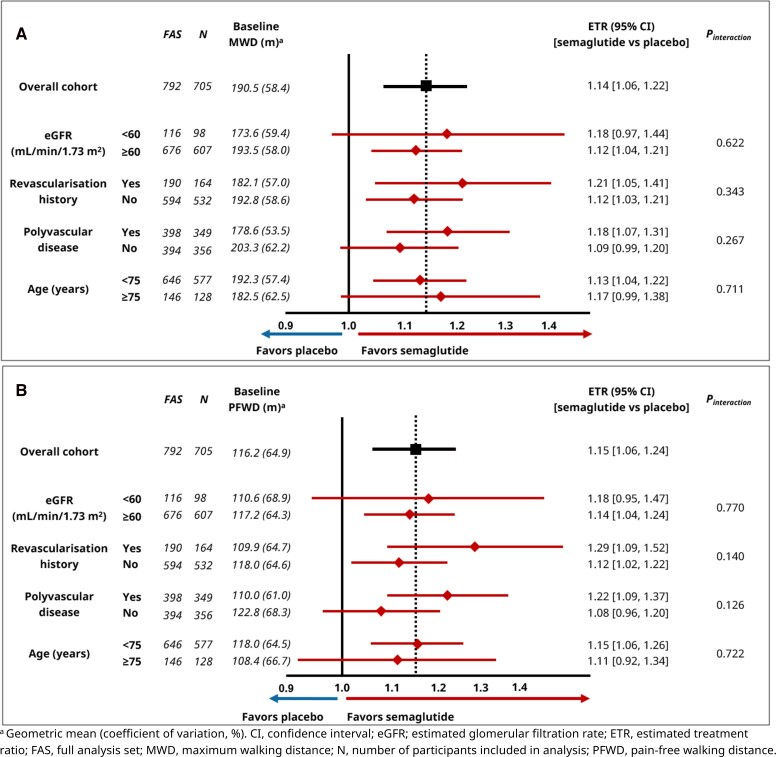
Primary endpoint, maximum walking distance (*A*), and confirmatory secondary endpoint, pain-free walking distance (*B*), measured at 52 weeks as ratio-to-baseline and presented as estimated treatment ratios

Of the 792 participants randomized, 195 (25%) were female and the overall mean (SD) age was 66.6 (9.4) years. Baseline walking capacities are presented in *Figure 1* and were comparable across treatment groups.^2^ Interaction *P*-values indicated consistent benefits of semaglutide vs placebo on walking capacity across subgroups, with stratified ETRs presented in *Figure 1*.

Treatment- and subgroup-specific ratios-to-baseline for the primary endpoint, MWD at week 52, were as follows (semaglutide/placebo): eGFR < 60 and ≥60 were 1.16/0.98 and 1.25/1.12, respectively; revascularization history ‘yes’ and ‘no’ were 1.26/1.04 and 1.23/1.11, respectively; polyvascular disease ‘yes’ and ‘no’ were 1.24/1.05 and 1.25/1.14, respectively; and age <75 and ≥75 years were 1.25/1.11 and 1.20/1.03, respectively.

The corresponding treatment- and subgroup-specific absolute improvements for MWD at week 52 were as follows (semaglutide/placebo): eGFR < 60 and ≥60 were 57.5/20.1 m and 83.7/45.0 m, respectively; revascularization history ‘yes’ and ‘no’ were 66.6/25.5 m and 84.3/43.7 m, respectively; polyvascular disease ‘yes’ and ‘no’ were 71.0/34.3 m and 89.3/48.1 m, respectively; and age <75 and ≥75 years were 84.9/47.2 m and 60.7/14.6 m, respectively.

## Discussion

Age and PAD severity independently worsen functional limitations and may alter responses to therapies targeting limb symptoms.^9^ Here we report that the benefits of semaglutide on walking capacity were consistent across clinical markers of PAD severity and prognosis, including impaired renal function, prior limb revascularization, polyvascular disease, and age.

Participants with impaired renal function realized similar benefits from semaglutide as those with normal function. Although semaglutide has been associated with greater weight loss in participants with lower vs higher eGFR,^7^ the modest weight reduction observed in STRIDE is unlikely to fully account for the observed functional improvement.^2^ Almost a quarter of the participants had previously undergone peripheral revascularization. People with PAD who have a history of revascularization have a greater risk of disease progression and adverse limb events than those without.^5,10^ Revascularization history thus represents a useful marker for adverse PAD limb events. Polyvascular disease is associated with increased likelihood of cardiovascular death, myocardial infarction, or stroke in people with PAD.^5^ In this analysis, neither markers appeared to modify the efficacy of semaglutide on functional improvements. Similar consistency was found across ages. Advanced age was associated with lower MWD and PFWD at baseline, but a consistent benefit across age groups supports that semaglutide may lead to improved quality of life and greater capacity for physical activity also in those beyond 75 years, for whom efficacy data for any PAD intervention is limited.

Several limitations are acknowledged. STRIDE was not designed, and thus sufficiently powered, to detect subgroup-treatment effects. However, reported interaction *P*-values support consistency, and the direction of all ETRs remains in favour of semaglutide. Smaller subgroup sample sizes are inherent with interaction analyses, and multiplicity adjustments were also not applied. With these factors in mind, the presented results should be interpreted as hypothesis-generating. The potential benefits of semaglutide should also be evaluated in people with more advanced PAD, who were excluded from STRIDE and typically bear greater functional impairment.

## Conclusion

Semaglutide consistently improved functional capacity in people with PAD and T2D regardless of age and across clinical markers of PAD severity. This new therapy meets a critical need in a high-risk population with few pharmaceutical alternatives available.

## Declarations

### Data Availability

Data will be shared with bona fide researchers upon reasonable request and following approval by an independent review board.

### Pre-registered Clinical Trial Number

The pre-registered clinical trial number is NCT04560998.

## References

[1] Nordanstig J, Behrendt CA, Baumgartner I, Belch J, Bäck M, Fitridge R, et al Editor's Choice – European Society for Vascular Surgery (ESVS) 2024 Clinical Practice Guidelines on the Management of Asymptomatic Lower Limb Peripheral Arterial Disease and Intermittent Claudication. Eur J Vasc Endovasc Surg 2024;67:9–96. 10.1016/j.ejvs.2023.08.067 37949800

[2] Bonaca MP, Catarig AM, Houlind K, Ludvik B, Nordanstig J, Ramesh CK, et al Semaglutide and walking capacity in people with symptomatic peripheral artery disease and type 2 diabetes (STRIDE): a phase 3b, double-blind, randomised, placebo-controlled trial. Lancet 2025;405:1580–93. 10.1016/S0140-6736(25)00509-4 40169145

[3] Rendell M, Cariski AT, Hittel N, Zhang P. Cilostazol treatment of claudication in diabetic patients. Curr Med Res Opin 2002;18:479–87. 10.1185/030079902125001245 12564659

[4] Hiatt WR, Fowkes FG, Heizer G, Berger JS, Baumgartner I, Held P, et al Ticagrelor versus clopidogrel in symptomatic peripheral artery disease. N Engl J Med 2017;376:32–40. 10.1056/NEJMoa1611688 27959717

[5] Kaplovitch E, Eikelboom JW, Dyal L, Aboyans V, Abola MT, Verhamme P, et al Rivaroxaban and aspirin in patients with symptomatic lower extremity peripheral artery disease: a subanalysis of the COMPASS randomized clinical trial. JAMA Cardiol 2021;6:21–9. 10.1001/jamacardio.2020.4390 32997098 PMC7527938

[6] Garimella PS, Hirsch AT. Peripheral artery disease and chronic kidney disease: clinical synergy to improve outcomes. Adv Chronic Kidney Dis 2014;21:460–71. 10.1053/j.ackd.2014.07.005 25443571 PMC4254470

[7] Cherney DZI, Hadjadj S, Lawson J, Mosenzon O, Tuttle K, Vrhnjak B, et al Hemoglobin A1c reduction with the GLP-1 receptor agonist semaglutide is independent of baseline eGFR. Kidney Int Rep 2022;7:2345–55. 10.1016/j.ekir.2022.07.167 36531884 PMC9751689

[8] Tamargo J, Kjeldsen KP, Delpón E, Semb AG, Cerbai E, Dobrev D, et al Facing the challenge of polypharmacy when prescribing for older people with cardiovascular disease. A review by the European Society of Cardiology working group on cardiovascular pharmacotherapy. Eur Heart J Cardiovasc Pharmacother 2022;8:406–19. 10.1093/ehjcvp/pvac005 35092425

[9] Houghton JSM, Saratzis AN, Sayers RD, Haunton VJ. New horizons in peripheral artery disease. Age Ageing 2024;53:afae114. 10.1093/ageing/afae114 38877714 PMC11178507

[10] Beckman JA, Schneider PA, Conte MS. Advances in revascularization for peripheral artery disease: revascularization in PAD. Circ Res 2021;128:1885–912. 10.1161/CIRCRESAHA.121.318261 34110904

